# Genome Sequence of *Thalassolituus oleivorans* MIL-1 (DSM 14913^T^)

**DOI:** 10.1128/genomeA.00141-13

**Published:** 2013-04-18

**Authors:** Peter N. Golyshin, Johannes Werner, Tatyana N. Chernikova, Hai Tran, Manuel Ferrer, Michail M. Yakimov, Hanno Teeling, Olga V. Golyshina, MAMBA Scientific Consortium

**Affiliations:** School of Biological Sciences, Bangor University, Bangor, Gwynedd, United Kingdoma;; Max Planck Institute for Marine Microbiology, Bremen, Germanyb;; Institute of Catalysis-CSIC, Madrid, Spainc;; Institute for Coastal Marine Environments-CNR, Messina, Italyd

## Abstract

*Thalassolituus oleivorans* is one of the most prevalent marine gammaproteobacteria in microbial communities, emerging after oil spills in coastal, estuarine, and surface seawaters. Here, we present the assembled genome of strain *T. oleivorans* MIL-1 (DSM 14913^T^), which is 3,920,328 bp with a G+C content of 46.6%.

## GENOME ANNOUNCEMENT

*Thalassolituus oleivorans* MIL-1 was isolated from an enrichment culture established from a seawater and sediment sample collected in the harbor of Milazzo, Sicily, Italy, from a depth of about 5 m, and was supplemented with tetradecane as the sole carbon source (1). The strain *T. oleivorans* is a typical representative of the so-called obligate hydrocarbonoclastic marine bacteria (OHCB) that are characterized by their ability to metabolize only a very restricted spectrum of carbon substrates (2). Like many other OHCB, such as *Alcanivorax* spp. (3), *Oleispira* spp., (4) and *Oleiphilus* spp. (5), *T. oleivorans* grows almost exclusively on aliphatic hydrocarbons and their derivatives, fatty acids and fatty alcohols. It is a sickle-shaped and motile bacterium that has been shown to play an important role in the biodegradation of crude oil and has been detected in marine environments worldwide, from Polar areas, such as the Barents Sea (Arctic Ocean) and Ross Sea (Antarctic coastal area), to warmer locations (Black and Mediterranean Seas), primarily in oxygenated surface waters and sediments and coastal and estuarine areas (6, 7).

Here, we report the genome sequence of *T. oleivorans* MIL-1. DNA was extracted using the G'NOME DNA extraction kit (MP Biomedicals, Santa Ana, CA) according to the manufacturer’s instructions. The genome was sequenced at Fidelity Systems Ltd. (Gaithersburg, MD) using a combination of Illumina short paired-end (400 bp) and long mate-paired (3,600 bp) insert libraries with a read length of 100 bases. Sequencing was undertaken according to the manufacturer’s protocols on an Illumina HiSeq 2000, with the modification that TopoTaq DNA polymerase was used for the PCR amplification of the fragmented genome library (8). A subset of one million reads of the short-insert library was assembled with Phrap to create initial contigs. Subsequent scaffolding, gap filling, and repeat resolution were performed using the Phred/Phrap/Consed software package (9) and Fidelity Systems’ in-house finishing software. The complete short-insert library data set was used to correct potential base errors and to assess the quality of the assembly. The error rate of the completed genome sequence is <1 in 100,000 (Phred 50). The final assembly contains 12,110,290 short-insert library reads and provides 294× coverage of the genome. The automated genome annotation was performed at Fidelity Systems Ltd., using FgenesB 2.0 (SoftBerry, Inc., NY). For annotation of RNA genes, the Rfam 11.0 database (http://rfam.sanger.ac.uk) (10) and the freeware Infernal 1.0.2 (http://infernal.janelia.org) (11) were used.

The genome comprises 3,603 protein-coding genes (1,565 hypothetical genes and 2,038 with annotated functions), four rRNA operons, two of which are clustered, and 49 tRNAs for all 20 amino acids; a selenocysteine lyase is also present. Peculiar regions in the genome comprise a possible prophage (genome position kb 506 to 542) and a large repeat region (position ~2.35 Mbp) in the direct vicinity of a cluster of Clustered Regularly Interspersed Short Palindromic Repeats (CRISPR) proteins.

### Nucleotide sequence accession number. 

The genome sequence of *T. oleivorans* MIL-1 (DSM 14913^T^) is available in GenBank/EMBL/DDBJ under the accession no. HF680312.
